# Evidence of new endemic foci of the foodborne helminths *Angiostrongylus* spp. in rats in selected communities in the Philippines

**DOI:** 10.1186/s13071-025-06989-4

**Published:** 2025-10-14

**Authors:** Allen Jethro I. Alonte, Martha E. Betson, Billy P. Divina, Vachel Gay V. Paller

**Affiliations:** 1 Institute of Biological Sciences, College of Arts and Sciences, University of the Philippines Los Baños, Los Baños, Laguna Philippines; 2 School of Veterinary Medicine, University of Surrey, Guildford, Surrey UK; 3 Department of Paraclinical Sciences, College of Veterinary Medicine, University of the Philippines Los Baños, Los Baños, Laguna Philippines

**Keywords:** *Angiostrongylus* spp., Angiostrongyliasis, Meningoencephalitis, Neglected tropical disease, Foodborne helminths

## Abstract

**Background:**

*Angiostrongylus cantonensis* (rat lungworm) is a zoonotic foodborne parasite causing meningoencephalitis among humans and animals. Neuroangiostrongyliasis is a globally emerging public health concern with several reported outbreaks; however, it remains neglected in the Philippines, where a lack of information on this parasite leads to underreporting and misdiagnosis. A good understanding of parasite epidemiology underpinned by accurate diagnosis is essential for treatment and control of parasitic diseases. Thus, this study aimed to determine the prevalence of *Angiostrongylus* spp. in rats in selected communities on Mindanao and Luzon Islands in the Philippines and provide accurate identification using molecular techniques.

**Methods:**

A total of 126 rats were collected from selected communities in Laguna, Davao del Sur, Agusan del Sur, and Surigao del Norte. Lungs were harvested after dissection and artificially digested to isolate the parasite. DNA was extracted from the parasite, and SSU-rRNA and COI genes were amplified and sequenced.

**Results:**

Results showed an *Angiostrongylus* spp. prevalence of 37.3% in rats with significantly higher prevalence in rural and sub-urban communities. Molecular analysis revealed two species: *Angiostrongylus cantonensis* and *A. malaysiensis*. This represents the first report of co-endemic *Angiostrongylus* spp. in Agusan del Sur, Mindanao.

**Conclusions:**

Our study revealed a high prevalence of *Angiostrongylus* spp. among rats from selected communities in the Philippines and identified new endemic sites, showing that the distribution of the parasite is wider than previously appreciated. Furthermore, two species were identified, which provides evidence of diverse *Angiostrongylus* species in the country. However, further studies are needed to investigate the pathogenicity of *A. malaysiensis*. Evidence of *Angiostrongylus* spp. in rats and the habit of eating raw or improperly prepared food in the surveyed communities may imply unseen transmission of *Angiostrongylus* spp. to humans. This highlights the need to establish the public health importance of angiostrongyliasis in the country starting with a surveillance scheme for this parasite.

**Graphical abstract:**

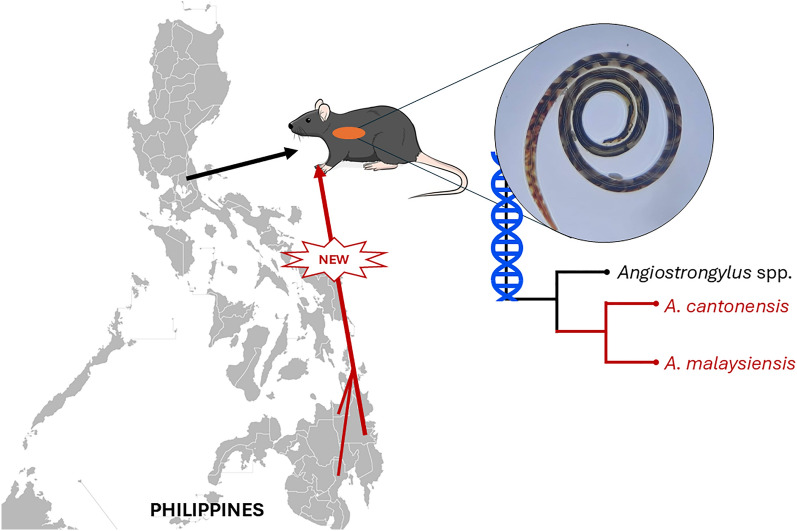

**Supplementary Information:**

The online version contains supplementary material available at 10.1186/s13071-025-06989-4.

## Background

Neuroangiostrongyliasis is an emerging infectious disease of medical and veterinary public health concern [1–4]. It is caused by the zoonotic nematode *Angiostrongylus cantonensis*, which in the adult stage inhabits the pulmonary artery of the rat definitive host. Infection may be acquired in humans through ingestion of raw or improperly prepared food harboring the third-stage larva of the nematode including snails, slugs, crustaceans, shrimps, planarians, and even vegetables [5]. There is also evidence of transmission through contaminated water [6].

Infection is associated with a diverse array of non-specific signs and symptoms. Human angiostrongyliasis caused by *A. cantonensis* is the leading cause of eosinophilic meningoencephalitis worldwide. This is related to the larval migration to the central nervous system where the parasite consequently dies, causing a cascade of immunological reactions often forming granulomas in the brain and sometimes leading to death [7]. Several outbreaks of human angiostrongyliasis have occurred in humans in different countries [8–13]. Similarly, cases of angiostrongyliasis in dogs have been reported in different parts of the world, attributed to the species *Angiostrongylus vasorum* [14–17]. On the other hand, there is no established evidence of the pathogenicity of *Angiostrongylus malaysiensis*, which is morphologically similar to *A. cantonensis.* However, *A. malaysiensis* DNA has been found along with *A. cantonensis* DNA in the cerebrospinal fluid of patients with eosinophilic meningitis [4].

Despite the serious consequences to human and animal health, angiostrongyliasis remains a neglected disease worldwide. There is a considerable lack of data on angiostrongyliasis in several known endemic countries [1–3], including the Philippines. Furthermore, diagnosis remains a challenge and recommendations for surveillance as well as standard treatment and management for angiostrongyliasis are yet to be formulated [10, 11].

*Angiostrongylus cantonesis* is considered an example of an emerging parasitic infection. It was first discovered in domestic rats from Guangzhou (formerly known as Canton), China [18], and since then it has been continually spreading to different parts of the world. Currently, *A. cantonensis* is known to be endemic in the Pacific Islands, Caribbean Islands, USA, Africa, Brazil, Spain, and Asia, including the Philippines [18–26]. Meanwhile, *A. malaysiensis* has been reported in Malaysia, Thailand, Laos, Myanmar, and Indonesia [2, 27].

Molecular studies on *Angiostrongylus* spp. isolated from different regions of the world showed several lineages, indicating multiple introductions of the parasite in an area. Molecular findings greatly contributed to distinguishing geographical isolates; however, phylogeographical patterns remain inconclusive because of the paucity of molecular data from other endemic countries such as the Philippines [24–37].

Studies on *Angiostrongylus* spp. in the Philippines reported prevalences as high as 100% in sampled rats or snails. However, these were limited to Luzon Island [38–42]. Additionally, there has been no molecular characterization of *Angiostrongylus* spp. in the country to date. Thus, this study aimed to determine the prevalence and molecular identity of *Angiostrongylus* spp. in rats present in selected communities on Luzon and Mindanao Islands in the Philippines.

## Methods

### Study sites and sampling design

**Fig. 1:**
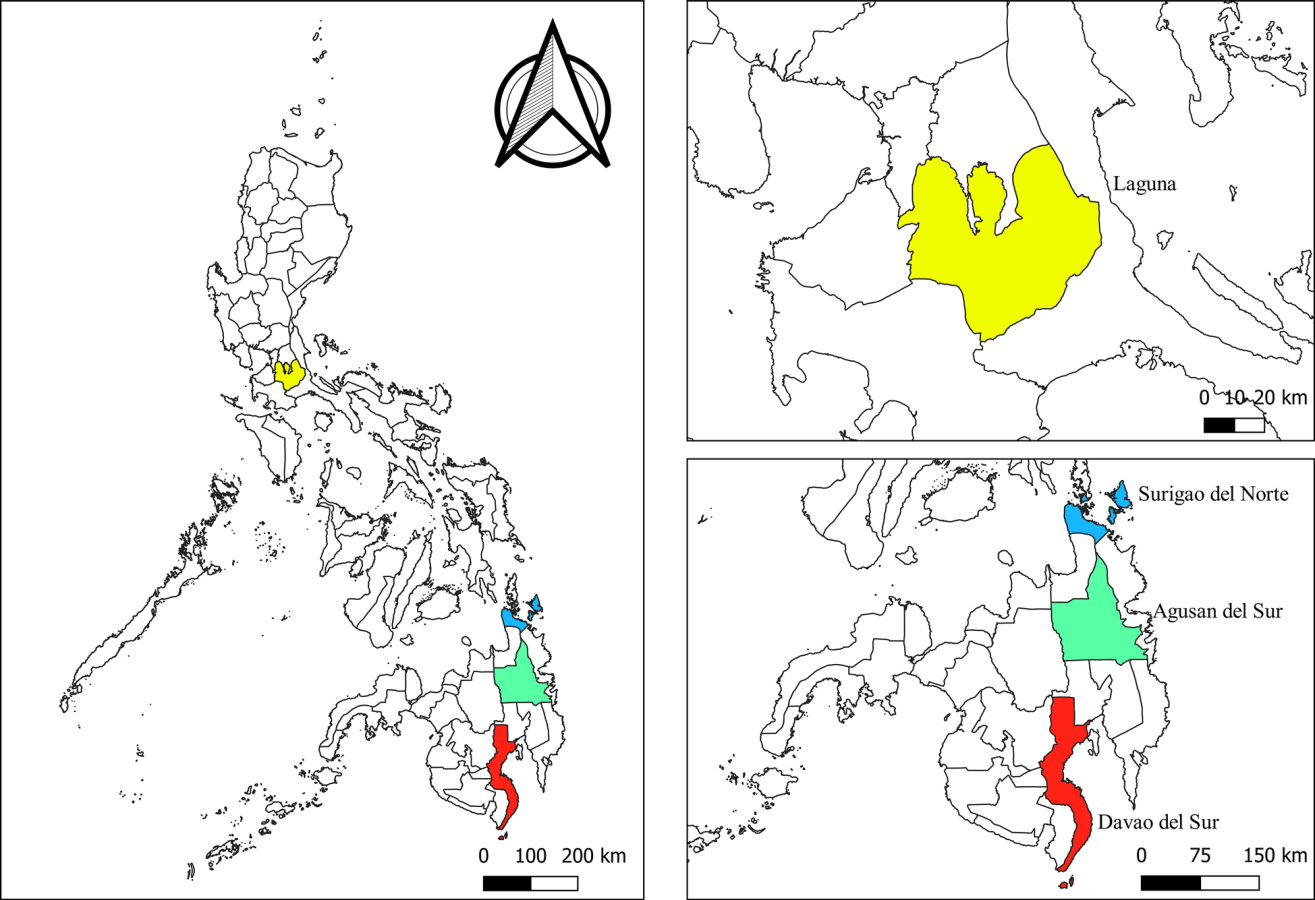
Map showing the sampling sites for rat collection in the Philippines. Left: the Philippines; top right: Laguna; bottom right: Agusan del Sur, Surigao del Norte, and Davao del Sur

This cross-sectional study was conducted in the provinces of Agusan del Sur, Davao del Sur, Laguna, and Surigao del Norte. Three of these provinces are on the Island of Mindanao where there has been limited surveillance for *Angiostrongylus* spp. in rodents to date (Fig. 1). Sites were selected based on sightings of rodents, evident close interaction between humans and rodents, and the known habit in communities of eating raw snails. Two communities from each province were included in the study. Table 1 summarizes the characteristics of the communities studied.

**Table 1:** Summary of the characteristics of the rat sampling sites in the Philippines

Characteristics	Communities			
	Agusan del Sur	Laguna	Davao del Sur	Surigao del Norte
Previous reports of *Angiostrongylus* spp.	X	**✓**	X	X
Evident close interaction of humans and rodents	**✓**	**✓**	**✓**	**✓**
Known habit of eating raw snails	**✓**	**✓**	**✓**	**✓**
Sightings of rodents	**✓**	**✓**	**✓**	**✓**
Near rice field areas	**✓**	**✓**	X	**✓**
Presence of irrigations or canals	**✓**	**✓**	**✓**	**✓**
Type of community	Rural	Sub-urban	Urban	Rural

A power analysis was used to determine the minimum sample size required for the study. Using a confidence level of 95%, margin of error of 5%, and power of 0.8, a minimum of 32 samples per community is required to observe the previously reported *A. cantonensis* prevalence of 100% in each area [38]. However, environmental permits limited collection to up to 30 rats per community resulting in a total of 240 rats for the four provinces.

### Rat trapping and dissection

Rat trapping was conducted from June 2019 to March 2023. Sample collection was semi-purposive in nature with live-catch traps set within a 100-m radius of households. Each trap was baited with approximately 3 g of roasted coconut coated with peanut butter [43]. In each community, ten (10) traps were set up in the afternoon (at around 5:00 to 6:00 p.m.), were geotagged, and were checked early in the morning (at around 5:00 to 7:00 a.m.).

Upon recovery of successful traps, initial visual inspections of animals trapped were done, and all animals other than *Rattus* spp. were immediately released. The genus *Rattus* was distinguished from other rodents by visual inspection [43–45]. Furthermore, as per the approved protocol of the Institutional Animal Care and Use Committee (IACUC), only adult rats were included in the study, and juvenile rats were immediately released upon inspection. Ensnared adult *Rattus* spp. still in the traps were then immediately transported to the field laboratory for immediate processing.

In the field laboratory, trapped rats were sedated using 5% isoflurane via drop jar method for easier handling and accurate body measurements. Sedated rats were gently removed from the traps and placed on a clean cloth for examination, including noting the fur color, sex, age, and body measurements. Rats were identified morphologically following descriptions from the previous literature [43–45].

Sedated rats were killed through intraperitoneal administration of 200 mg/kg pentobarbital sodium using a G26 needle syringe. The rats were dissected, and the lungs and heart were collected. These organs were individually placed in a resealable plastic bag, labeled with a unique identifier, and immediately stored at – 20 ℃ until further processing. The rat carcasses were disposed of by burying.

### Adult worm collection

The collected lungs and heart were grossly examined for adult *Angiostrongylus* spp. worms. Frozen organs were thawed and placed in a petri dish. The lungs were squashed in two petri plates and visually examined for adult worms under a stereoscope. Moreover, the lungs were carefully dissected to reveal the pulmonary arteries and check for the presence of adult worms [18]. Similarly, the heart was cut open to examine the inside of the heart valves [18]. Adult worms, if present, were collected and individually placed in a 2-ml microcentrifuge tube containing ~ 95% ethyl alcohol for preservation prior molecular analysis. Adult *Angiostrongylus* spp. worms were morphologically identified following the description of Bhaibulaya [46].

### Microscopic examination

After gross examination, the lungs and heart were digested using an artificial gastric juice to check for the presence of larvae. Artificial gastric juice was prepared in a 1-l beaker containing 500 ml distilled water and was placed on a hot plate with a magnetic stirrer and with temperature set at room temperature (~ 20–25 ℃). One gram of pepsin powder was added to the water followed by 10 ml concentrated hydrochloric acid. The solution was mixed continuously until all pepsin powder was completely dissolved, which usually takes around 3 to 5 min. Finally, distilled water was added to the solution to bring the final volume to 1-l. The prepared artificial gastric juice was stored at 4 ℃ and could be used for up to 1 week.

Subsequently, the organs were cut into smaller pieces to facilitate digestion and placed in a 250-ml beaker. About 100 ml artificial gastric juice was then added. The mixture was placed on a hot plate with a magnetic stirrer and the temperature set at 37 ℃ to simulate basal body temperature. The mixture was continuously stirred for 2 h or until all tissues were completely digested. The digested solution was filtered through a 250-µm sieve. The filtrate was placed in 50-ml conical tubes and centrifuged at 12,000 × g for 10 min. Some samples required more than one conical tube. After centrifugation, the supernatant was carefully decanted. Sediments were placed in a single conical tube, and 95% ethyl alcohol was added for preservation. The sediments were then viewed under a microscope for the presence of *Angiostrongylus* spp. larvae following descriptions of previous studies [4, 18].

### Molecular identification

Genomic DNA was extracted from all 16 adult *Angiostrongylus* spp. worms collected. Prior to extraction, the ethanol-preserved worm tissue was prepared by sectioning approximately 2 cm of the worm using a scalpel near the anterior part while carefully avoiding the reproductive organs. The sectioned tissue was washed thrice with phosphate-buffered solution to remove ethanol preservative, which may interfere during DNA extraction. DNA was extracted using DNeasy Blood and Tissue Kit (Qiagen, USA) following the manufacturer’s protocol. DNA was labeled and stored at − 20 °C until further use. The nuclear SSU-rRNA gene and mitochondrial COI gene were amplified for molecular characterization through conventional PCR using previously published primers (Table 2) [24].

**Table 2:** Primers used for amplification of target genes of *Angiostrongylus* spp.

Target genes	Primers
SSU-rRNA	Forward: 5′-AAAGTTAAGCCATGCATG-3′
	Reverse: 5′-CATTCTTGGCAAATGCTTTCG-3′
COI	Forward: 5′-TTTGTTTTGATTTTTTGGTC-3′
	Reverse: 5′-AGGATAAATCTAAATACTTACGAGGA-3′

PCR amplification for both genes was carried out in a 20-µl reaction mixture consisting of 10 µl Q5 High-Fidelity 2X master mix (New England Biolabs, UK), 1.6 µM primers, 2.6 µl nuclease-free water, and 1 µl DNA template. Negative control set-up using nuclease-free water as template was included in each run. The amplification profiles used in this study followed previously published protocols [24]. All samples showing the expected band size in the gel electrophoresis were confirmed through sequencing. Forward and reverse amplicon sequencing using the Sanger method was outsourced to Apical Scientific Laboratory (Selangor, Malaysia). Sequences generated were submitted to GenBank (PQ415441–PQ415547 and PQ415790–PQ415805).

### Data processing and analysis

The prevalence of *Angiostrongylus* spp. among collected rats was computed. Significant differences of prevalence across sites, sex, and host species were determined using the chi-squared test. Generalized mixed model analysis was also conducted to determine the effect of sex and host species on risk of *Angiostrongylus* spp. infection. The provinces were considered as a random effect in the models. In all analyses, a *p*-value < 0.05 was considered significant. Analyses were performed using R Statistical Software (v4.2.3) [47].

Initial analysis of nucleotide sequence data involved searches of NCBI nucleotide sequence databases using the BLAST algorithm to identify most closely related sequences. Multiple sequence alignment of the nucleotide sequences was then performed using the Clustal W algorithm [47] in the Geneious Prime software (v 2023.1.2; https://www.geneious.com) with all of the parameters set to default. The alignments were visually inspected, and leading and trailing misaligned regions were trimmed to minimize the effects of sequence length in the phylogenetic analysis. Phylogenetic trees were reconstructed separately for SSU-rRNA and COI genes. In both trees, the distantly related strongyles, *Metastrongylus salmi* and *Aelustrongylus abstrusus*, were used as outgroups. The best nucleotide substitution model was identified using jModelTest (v2.1) [48], and the substitution model with the lowest corrected Akaike information criterion (AICc) was selected. Hasegawa-Kishino-Yano model was used as the nucleotide substitution model for SSU-rRNA gene with gamma distribution set at 0.1010 as shape parameter (AICc = 3068.00), while the Tamura-Nei model with gamma distribution set at 0.4210 as shape parameter (AICc = 4099.40) was used for COI gene.

Maximum likelihood trees were constructed using PhyML (v3.0; Guindon et al., 2010) following the parameters set by the model identified. Maximum parsimony and neighbor-joining trees were constructed using PAUP* (v4.0; Swofford, 2003). Maximum parsimony followed a heuristic search strategy through random addition of sequences with 50 replications for each run. In each method, a bootstrap consensus tree was built with 5000 bootstrap replicates, and branches corresponding to partitions reproduced in < 50% bootstrap replicates were collapsed. Furthermore, Bayesian inference of phylogeny was also computed through determining the posterior probability (BPP). This was done using the Markov Chain Monte Carlo (MCMC) algorithm in MrBayes (v3.2) [50]. Parameters were set following the previously identified best substitution model. MCMC was run for 2 million generation sampling for every 200th generation and setting 500,000 (25% of the generations) as burn in. The number of generations was set to ensure convergence of the model with a split frequency value of < 0.01. Trees and statistics were summarized using the **sumt** and **sump** command, respectively.

Consensus trees generated were saved in newick format and was visualized using FigTree (v1.4.4) [50] and further enhanced using an image editor. Furthermore, haplotype network analyses in the form of minimum spanning networks [52] were constructed separately for COI sequences of *A. cantonensis* and *A. malaysiensis* using PopART (http://popart.otago.ac.nz) [53]. These networks were constructed to further explore relationship patterns among the different geographic isolates.

## Results

A total of 126 rats were collected, reaching 52.5% of the target sample size. Four species of the genus *Rattus* were identified: *R. argentiventer*, *R. exulans*, *R. norvegicus*, and *R. tanezumi*. Among the collected rats, *R. norvegicus* was the most commonly collected species with 42 individuals, followed by *R. tanezumi* (39), *R. argentiventer* (33), and lastly *R. exulans* (12). Moreover, among the collected rats, 75 were males and 51 were females (Table 3).

**Table 3:** Prevalence of *Angiostrongylus* spp. in rats in selected communities in the Philippines

	No. of rats collected *n*	Prevalence no. positive (%)	95% confidence interval	χ2	*p*-value	Mean intensity	Standard error
Communities							
Agusan del Sur	48	38 (79.17)	67.68–90.66	61.804	< 0.0001^*^	29.08	± 9.69
Davao del Sur	35	0					
Surigao del Norte	26	5 (19.23)	4.08–34.38			19.40	± 16.43
Laguna	17	4 (23.53)	3.36–43.69			133.00	± 54.51
*Rattus* spp.							
*R. argentiventer*	33	10 (30.30)	14.62–45.98	27.399	< 0.0001^*^	44.40	± 35.04
*R. exulans*	12	9 (75.00)	50.50–99.50			23.00	± 8.23
*R. norvegicus*	42	5 (11.90)	2.11–21.70			106.6	± 111.35
*R. tanezumi*	39	23 (58.97)	43.54–74.41			23.91	± 6.05
Sex							
Male	75	28 (37.33)	26.39–48.28	0.000	1.0000	36.68	± 15.92
Female	51	19 (37.25)	23.99–50.52			37.21	± 8.09
Overall	**126**	**47** **(37.30)**	**28.86–45.75**				

^*^Significant at 5% level

The overall observed prevalence of *Angiostrongylus* spp. among collected rats in the communities was 37.30%. Communities studied in Agusan del Sur had the highest observed prevalence with 79.17%, which was found to be significantly different compared to other communities (*p* < *0.001*). This was followed by Laguna with 23.53% and Surigao del Norte with 19.23%. Highest mean intensity was found in Laguna followed by Agusan del Sur and Surigao del Norte. No *Angiostrongylus* spp. were observed in rats collected from Davao del Sur (Table 3).

Among *Rattus* spp., *R. exulans* was found to have the highest infection prevalence with *Angiostrongylus* spp. at 75.0% (9/12) followed closely by *R. tanezumi* at 59.0% (23/39), *R. argentiventer* at 30.3% (10/33), and *R. norvegicus* at 11.9% (5/42). The prevalence found in *R. norvegicus* was significantly different compared to *R. tanezumi* and *R. exulans* (*p* < *0.05*). Moreover, there was no significant difference in the prevalence in male rats at 37.22% compared with females at 37.25 (*p* > *0.05*) (Table 4). Results of mixed model analysis showed no significant factors contributing to the risk of having *Angiostrongylus* spp. in rats (Table 4).

**Table 4:** Results of the binomial generalized mixed model analysis for risk factors related to *Angiostrongylus* spp.

	Coefficient	Standard error (SE)	z-value	*p*-value
Fixed effects:				
Intercept	− 1.5420	0.778	− 1.980	0.048
Species (baseline *Rattus argentiventer*)				
*R. exulans*	0.1042	0.466	0.223	0.823
*R. norvegicus*	− 0.8376	0.776	− 1.080	0.280
*R. tanezumi*	0.0089	0.382	0.023	0.981
Sex (baseline female)				
Male	0.0236	0.304	0.078	0.938
Random effects				
	**Variance**	**SE**		
Communities	1.591	1.262		
Model fit				
	**Deviance**	**AIC**^**a**^	**BIC**^**b**^	
	153.1	167.1	187.0	

^*^Significant at 5% level

^*a*^*Akaike information criterion*

^*b*^*Bayesian information criterion*

Molecular analysis of sampled *Angiostrongylus* spp. worms was then conducted. Genomic DNA was extracted from 16 adult worms obtained from 7 rats in Laguna (11 worms) and Agusan del Sur (5 worms). The SSU-rRNA gene and COI gene were successfully amplified from all 16 worms. The length of SSU-rRNA gene generated from this study ranged from 803 to 839 bp, while for the COI gene, it ranged from 387 to 436 bp. SSU-rRNA and COI sequences were successfully generated from all 16 worms. Comparison with existing sequences in the NCBI databases indicated that all sequences from Laguna have 100% similarity with *A. cantonensis*, while in Agusan del Sur sequences share 100% similarity with *A. malaysiensis* (4 worms) and *A. cantonensis* (1 worm) (see Supplementary Table 1 and Supplementary Table 2).

The generated SSU-rRNA sequences were aligned with published SSU-rRNA sequences using the CLUSTAL W algorithm [47]. Aligned sequences showed 785 monomorphic sites and 20 polymorphic sites. Among the monomorphic sites, one conserved DNA region is seen at position 192 to 384 with a conservation and homozygosity value of 1.000 (*p* = *0.0039*).

**Fig. 2:**
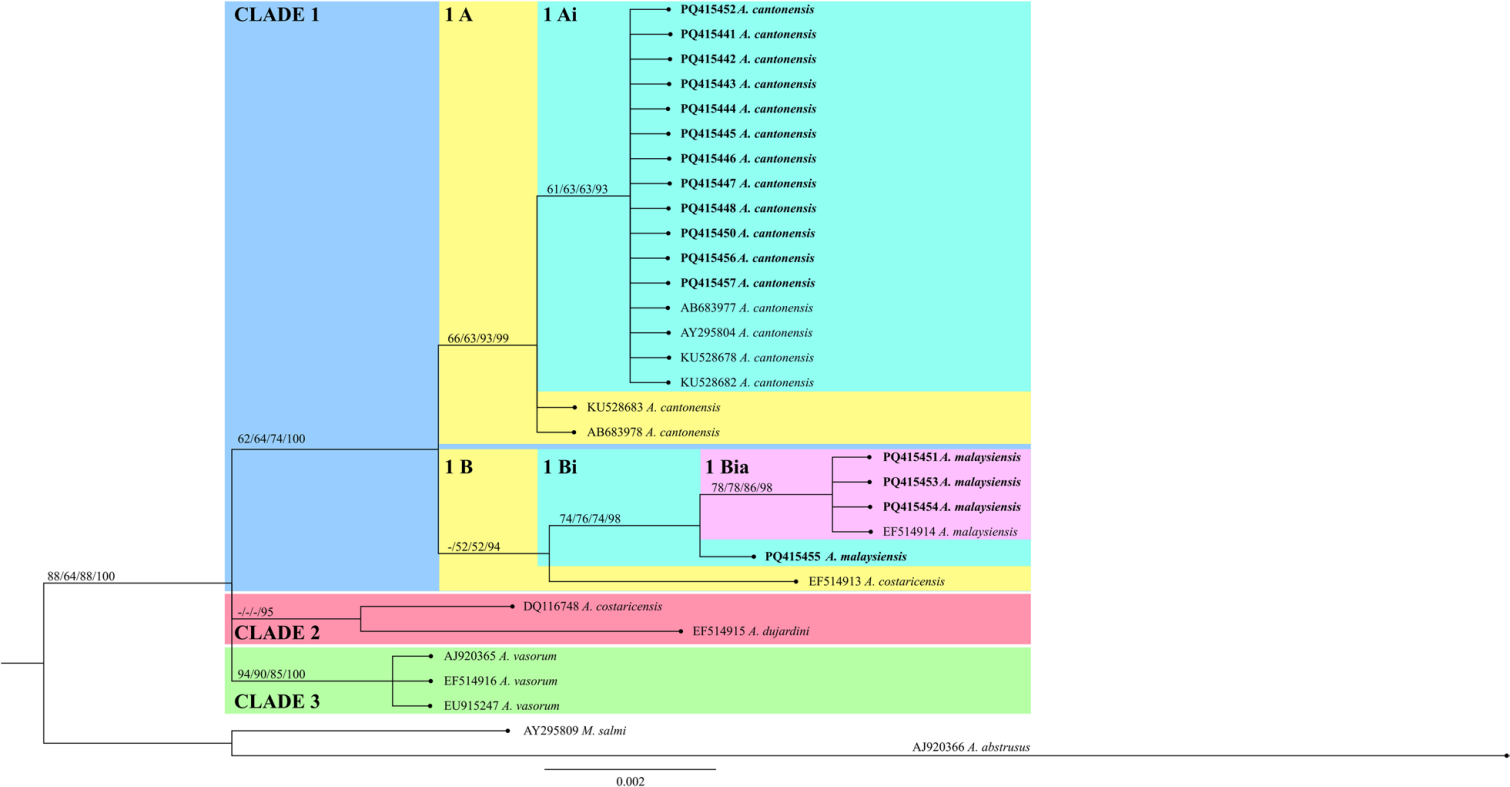
Phylogenetic tree of *Angiostrongylus* spp. based on the SSU-rRNA gene. Maximum likelihood, maximum parsimony, and neighbor-joining bootstrap support values as well as Bayesian posterior probability were indicated on each branch inside parentheses (ML/MP/NJ/BPP). Branch values < 50% were collapsed. Boldface text indicates sequences generated in this study

Figure 2 shows the phylogenetic tree based on SSU-rRNA genes of *Angiostrongylus* spp. reconstructed using the aligned sequences. The tree based on SSU-rRNA gene formed three major clades. The *A. cantonensis* isolates from the Philippines grouped with *A. cantonensis* genotype 1. The branch merged with the *A. cantonensis* G2 forming subclade 1A, where all *A. cantonensis* sequences are found. Furthermore, the *A. malaysiensis* sequences generated from this study formed the subclade 1Bia with high branch support value. This branch merged with ADS 5, forming the subclade labeled 1Bi.

For COI gene, aligned sequences showed 192 monomorphic sites, 124 polymorphic sites, and one gap. Among the monomorphic sites, two conserved DNA regions were seen, one at position 58 to 184 with a conservation value of 0.701 and homozygosity value of 0.934 (*p* = 0.0037) and another at 116 to 185 with a conservation value of 0.700 and homozygosity of 0.942 (*p* = 0.0476).

**Fig. 3:**
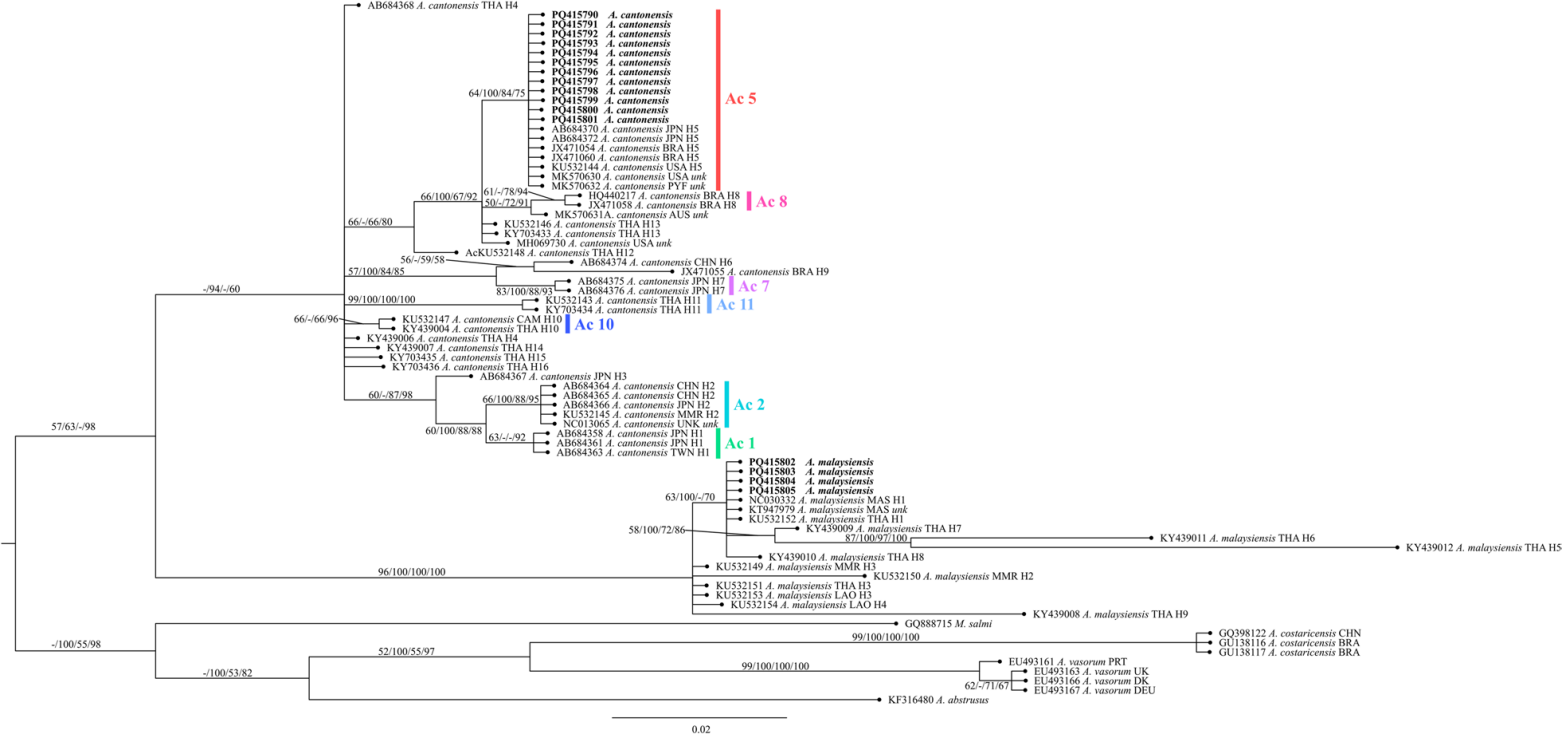
Phylogenetic tree of *Angiostrongylus* spp. based on the COI gene. Maximum likelihood, maximum parsimony, and neighbor-joining bootstrap support values as well as Bayesian posterior probability were indicated in each branch inside parentheses (ML/MP/NJ/BPP). Branch values < 50% were collapsed. Boldface text indicates sequences generated in this study

Figure 3 shows the phylogenetic tree based on COI genes of *Angiostrongylus* spp. reconstructed using the aligned sequences. The tree based on the COI gene formed separate clades for *A. cantonensis* and *A. malaysiensis*. The *A. cantonensis* sequences generated in this study formed a single group together with sequences from Japan, Brazil, the USA, and French Polynesia. With the exception of sequences with accession numbers MK570630 and MK570632, all of the sequences from the database were identified as haplotype 5. This formed the subclade labeled Ac5. Furthermore, *A. cantonensis* sequences from Brazil identified as haplotype 8 formed the distinct subclade Ac8. This merged with the Australian *A. cantonensis* sequence, which at present has an unknown haplotype. Subclades Ac5 and Ac8 formed a paraphyletic grouping with *A. cantonensis* sequences from Thailand identified as haplotype 13 and an *A. cantonensis* sequence from the USA with unknown haplotype. This merged with *A. cantonensis* haplotype 12 sequence from Thailand. The *A. malaysiensis* sequences from this study grouped with isolates from Malaysia and Thailand identified as haplotype 1 and another Thailand isolate identified as haplotype 8.

**Fig. 4:**
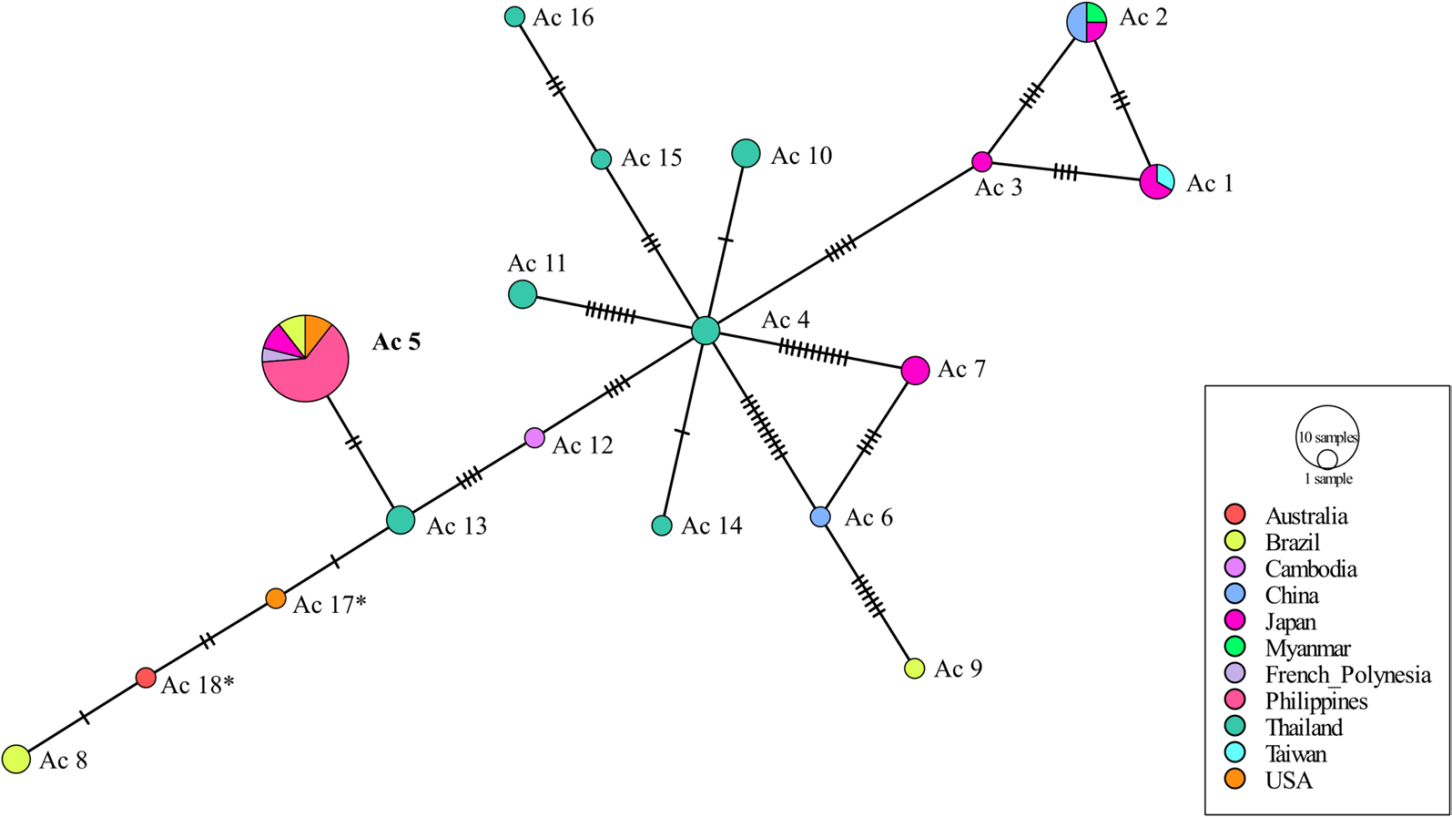
Haplotype network in the form of minimum spanning network based on the *Angiostrongylus cantonensis* COI sequences. One hatch mark = one base mutation difference; size of the circle is related to the number of sequences included in each group; *previously unknown haplotypes. Boldfaced text = haplotype of the sequences generated in the present study

**Fig. 5:**
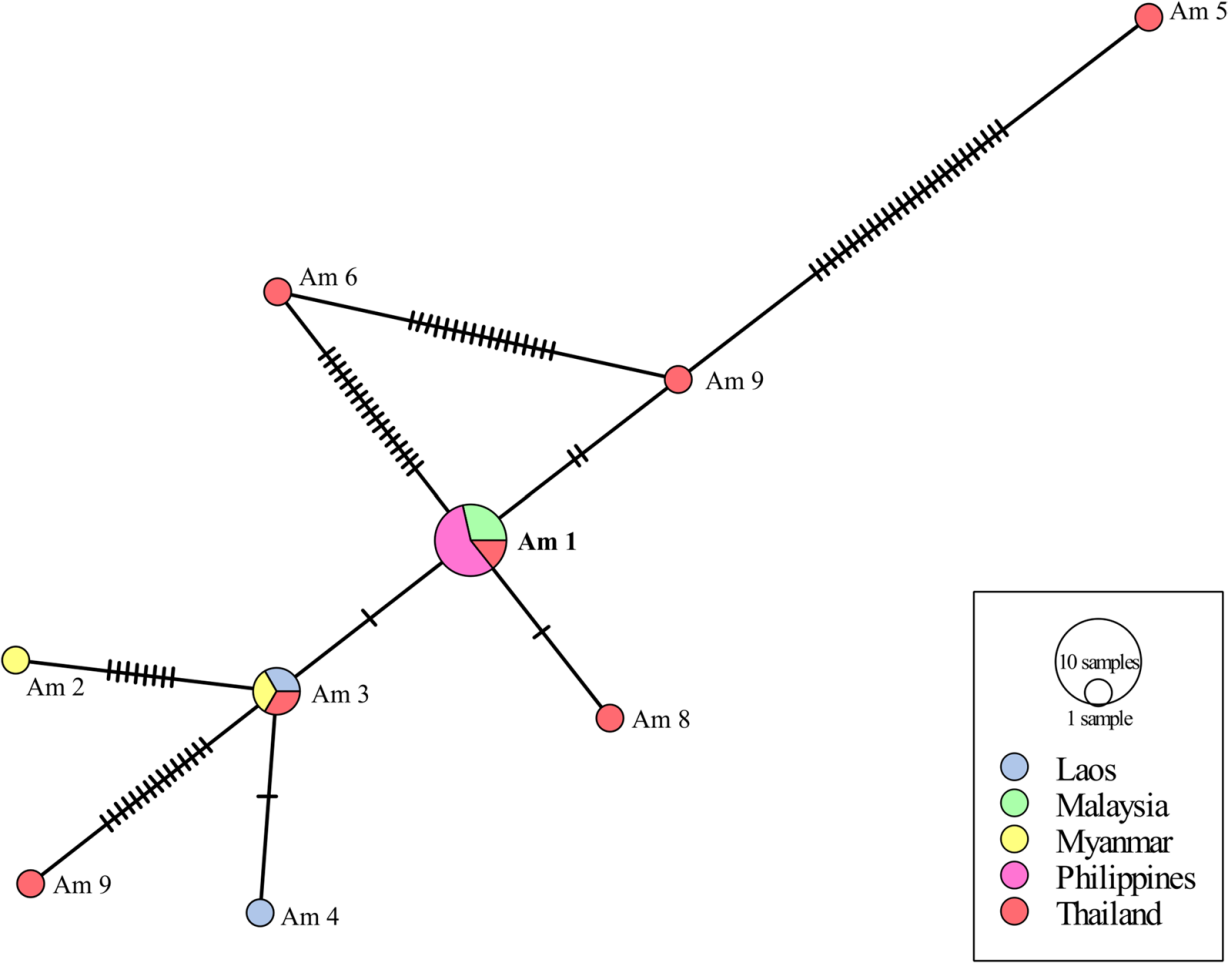
Haplotype network in the form of minimum spanning network based on the *Angiostrongylus malaysiensis* COI sequences. One hatch mark = one base mutation difference. Size of the circle is related to number sequences included in each group; *Previously unknown haplotypes. Boldfaced text = haplotype of the sequences generated in the present study

Figure 4 shows the haplotype network based on the COI sequences of *A. cantonensis*. It can be seen that *A. cantonensis* isolates from the Philippines are identical to those isolates in French Polynesia, Japan, Brazil, and the USA identified as haplotype Ac 5. Figure 5 shows a minimum spanning network based on the COI sequences of *A. malaysiensis* haplotypes from several parts of the world plus the sequences generated in this study. Similar to *A. cantonensis*, the diversity of the *A. malaysiensis* population appears to be highest in Thailand, where all haplotypes except for two—Am 2 and Am 4—have been identified. This is followed by Myanmar and Laos with two haplotypes each with Am 2 and Am 3 for Myanmar and Am 3 and Am 4 for Laos. In Malaysia and the Philippines, only Am 1 was seen.

## Discussion

The emerging pathogens *A. cantonensis* and *A. malaysiensis* show wider distribution than previously reported. However, this remains neglected in several endemic countries, as evident in the lack of baseline data with which to develop policy recommendations. Recent studies in the Philippines are currently limited to the island of Luzon. Hence, this present study included communities from Mindanao where the habit of eating raw food such as snails is a part of the traditional norm in some local communities.

Results of the study showed an overall prevalence of 37.30% in rats in the selected communities. Castillo and Paller [38] found a higher prevalence at 100% among rats in the Philippines, while results of Tujan et al. [42] and Estaño et al. [40] found lower prevalence ranging from 24.4% to 31%. In this study, significantly higher prevalence was reported in Agusan del Sur (79.17%) followed by Laguna (23.53%) and Surigao del Norte (19.23%). The results of mixed model analysis showed that neither the species nor sex of the rat definitive host is a significant factor contributing to the risk of infection. This may imply that rats, regardless of sex and species, are equally at risk for *Angiostrongylus* spp.

This study is the first to report *Angiostrongylus* spp. in rats from the island of Mindanao specifically in Agusan del Sur and Surigao del Norte. No *Angiostrongylus* spp. were found in rats from Davao del Sur. This does not necessarily imply the absence of the parasite in the province. Expanding surveillance efforts through inclusion of intermediate hosts as well as use of more sensitive detection tools are necessary to confirm the absence of the *Angiostrongylus* spp. in the area [54].

The prevalence in Laguna reported in this study is similar to the previous report in the province at 24.4% [40]. This shows the continuing circulation of *Angiostrongylus* spp. in this region and suggests that there is a lack of awareness of the parasite, meaning that necessary control measures are not being undertaken. Meanwhile, the variability in prevalence across the communities may be due to differences in the ecology of each area as well as focal distribution of the parasite in the area. Previous studies in the Philippines reported high prevalence of *Angiostrongylus* spp. in rural areas compared to urban areas, which could be related to the distribution of the snail intermediate host of *Angiostrongylus* spp., which usually inhabits rice fields [38, 40, 42]. The overlap between the distribution of the rat definitive host and snail intermediate host supports the continued life cycle of *Angiostrongylus* spp. in the country. Similarly in this study, the highest prevalence was observed in Agusan del Sur where the selected communities in the province are rural and are characterized by presence of rodents, rice field areas, and irrigations or canals, which may support the life cycle of *Angiostrongylus* spp.

Similar to other parasites with indirect life cycles, focal distribution may also be observed for *Angiostrongylus* spp. since this parasite requires a snail intermediate host. Differences in the distribution of snail intermediate hosts influence the prevalence of *Angiostrongylus* spp., which can vary greatly across an environmental gradient [55]. Moreover, prevalences ranging from 1% to 18.27% among snail intermediate hosts collected in agricultural areas in the Philippines were previously reported [39, 42]. This highlights the need to include snail intermediate hosts in future studies to completely elucidate the effects of the environment to the distribution of rat lungworm in the Philippines.

Although focal distribution is observed at present, the spread of endemicity may be expected in the future due to human and animal movement as well as climate change leading to flooding and typhoons [56, 57]. These events can facilitate the distribution of infected hosts to non-endemic areas. Climate change studies may be necessary to completely map the endemicity of *Angiostrongylus* spp. and predict the spread of the parasite [54–57].

In the Philippines, molecular studies have been limited to molecular identification using the SSU-rRNA gene [41, 42]. In other endemic countries, several molecular markers including SSU-rRNA, ITS, COI, 66 kDa protein, and even the whole mitogenome have been used to molecularly characterize *Angiostrongylus* spp. [24–37]. To our knowledge, this is the first study to characterize *Angiostrongylus* spp. in the Philippines using SSU-rRNA and COI genes.

Results showed that two species are present in the selected communities: *A. cantonensis* and *A. malaysiensis*. In Laguna, adult worms show high similarity with *A. cantonensis*, confirming the previous reports in the area as identified microscopically. On the other hand, overlapping endemicity of *A. cantonensis* and *A. malaysiensis* is observed in Agusan del Sur, which is also observed in other Asian countries [27]. These two species share several similarities particularly in their life cycle and morphology [5, 46]. Studies show morphological differences between the adult worms are largely overlapping and may be inconclusive in different stages of the life cycle or sex of the parasite [18, 46, 58]. Among these two species, *A. cantonensis* is known to be more pathogenic, causing neuorangiostrongyliasis [10–13], while further studies are needed to establish the pathogenicity of *A. malaysiensis* [4]. Nevertheless, the detection of the two species in Agusan del Sur implies diverse *Angiostrongylus* spp. in this area and possibly in other parts of the country, which may also lead to development of hybrids [58]. One limitation of the study is that no sequences were generated from parasites isolated in Surigao del Norte. An attempt was made to sequence the larvae obtained from rats collected in this region. However, no clean and interpretable sequence reads were generated despite repeated sequencing.

The *A. cantonensis* COI sequences generated from this study clustered with *A. cantonensis* isolated from Japan, Brazil, the USA, and French Polynesia. This is observed in the phylogenetic tree as well as the haplotype network. Furthermore, the *A. malaysiensis* COI sequences generated in this study are identical to those isolated from Malaysia and Thailand. It was also observed that the mitochondrial COI gene showed greater variation, which proved to be particularly useful in studying intraspecific variation [59, 60]. The phylogenetic tree as well as haplotype network based on the mitochondrial COI gene showed variation for *A. cantonensis* and *A. malaysiensis*. Using this gene, 18 haplotypes were observed for *A. cantonensis*—two of which were previously unidentified (MK570631 and MH069730)—while nine haplotypes were identified for *A. malaysiensis*. The *A. cantonensis* isolates from the Philippines showed high similarity with haplotype Ac 5, while for *A. malaysiensis*, haplotype Am 1 was very similar to parasites from the Philippines. However, whether phenotypic differences such as antigenicity exist for these haplotypes remains unexplored [61].

The present study utilized SSU-rRNA and COI genes. The SSU-rRNA, although it has little intraspecific variation, is found to be useful in analyzing interspecific relationships and can be used to identify the species of *Angiostrongylus* spp. [2, 21, 24, 27]. Moreover, complimenting the SSU-rRNA with COI helped in studying intraspecific variations of *Angiostrongylus* spp. [21, 57, 58]. Furthermore, the study used published primers that only amplify partial regions of both genes, thus limiting the variations observed. Nevertheless, previous studies showed that most variation in these genes can be seen in the 5’ half of the gene regions [41, 61]. As such, partial regions are still informative, although studies in the future may require complete sequences, additional gene markers, or even whole genome sequences to elucidate more information about *Angiostronglyus* spp. in the country [28, 62].

## Conclusions

The high prevalence of *Angiostrongylus* spp. observed among rats from selected communities in the Philippines shows that the distribution of *Angiostrongylus* spp. is wider than previously known. To our knowledge, this is the first study to report *Angiostrongylus* spp. in Agusan del Sur and Surigao del Norte. Furthermore, the evidence of both *A. cantonensis* and *A. malaysiensis* among *Rattus* spp. in Agusan del Sur provides proof of diverse *Angiostrongylus* spp. in the Philippines. The lack of surveillance studies on angiostrongyliasis in the country may mean that human cases of this parasitic infection are severely underreported, leading to neglect. Although there are no reported cases of human angiostrongyliasis in the country yet, it is more likely to be missed or misdiagnosed because of lack of guidance by way of policy for clinical practitioners to even consider the disease. The presence of *Angiostrongylus* spp. in rat definitive hosts shows evidence of the endemicity of the parasite in the country. This together with the habit of eating raw or improperly prepared food such as snails or vegetables as part of traditional or cultural norms puts humans at higher risk of contracting this seriously debilitating infection. This highlights the need for action to control of *Angiostrongylus* spp. in the country starting by developing a surveillance scheme for the parasite.

## Supplementary Information

Additional file 1.

## Data Availability

The data that support the findings of this study are reported in the study. Sequence data that support the findings of this study have been deposited in GenBank with primary accession codes PQ415441–PQ415547 and PQ415790–PQ415805.
