# Supplementary material for: Evidence of new endemic foci of the foodborne helminths Angiostrongylus spp. in rats in selected communities in the Philippines
Source: Parasit Vectors. 2025 Oct 14;18:408. doi: 10.1186/s13071-025-06989-4 (PMC12522548; doi:10.1186/s13071-025-06989-4)
Supplement: Supplementary file 1 — Additional file 1. [file 13071_2025_6989_MOESM1_ESM.docx]

Supplementary Table 1. Results of BLAST searches of NCBI databases with DNA sequences of the SSU-rRNA gene from *Angiostrongylus* spp. adult worms collected from rats in Laguna and Agusan del Sur

| **Site/Code** | | **Length (bp)** | **Details of the top BLAST hits** | | | | |
| --- | --- | --- | --- | --- | --- | --- | --- |
|  |  |  | **Identity** | **% Similarity** | **Query Cover** | **E-value** | **Accession Number** |
| **Laguna** | | | | | | | |
| 1 | PQ415441 | 837 | *A. cantonensis* | 100% | 99% | 0.0 | AY295804 |
| 2 | PQ415442 | 839 | *A. cantonensis* | 100% | 100% | 0.0 | AY295804 |
| 3 | PQ415443 | 803 | *A. cantonensis* | 100% | 99% | 0.0 | AY295804 |
| 4 | PQ415444 | 834 | *A. cantonensis* | 100% | 100% | 0.0 | AY295804 |
| 5 | PQ415445 | 834 | *A. cantonensis* | 100% | 100% | 0.0 | AY295804 |
| 6 | PQ415446 | 834 | *A. cantonensis* | 100% | 100% | 0.0 | AY295804 |
| 7 | PQ415447 | 835 | *A. cantonensis* | 100% | 100% | 0.0 | AY295804 |
| 8 | PQ415448 | 835 | *A. cantonensis* | 100% | 100% | 0.0 | AY295804 |
| 9 | PQ415450 | 835 | *A. cantonensis* | 100% | 100% | 0.0 | AY295804 |
| 10 | PQ415456 | 835 | *A. cantonensis* | 100% | 100% | 0.0 | AY295804 |
| 11 | PQ415457 | 837 | *A. cantonensis* | 100% | 100% | 0.0 | AY295804 |
| **Agusan del Sur** | | | | | | | |
| 12 | PQ415451 | 838 | *A. malaysiensis* | 100% | 99% | 0.0 | EF514914 |
| 13 | PQ415452 | 837 | *A. cantonensis* | 100% | 99% | 0.0 | AY295804 |
| 14 | PQ415453 | 838 | *A. malaysiensis* | 100% | 99% | 0.0 | EF514914 |
| 15 | PQ415454 | 838 | *A. malaysiensis* | 100% | 99% | 0.0 | EF514914 |
| 16 | PQ415455 | 838 | *A. malaysiensis* | 100% | 99% | 0.0 | EF514914 |
|  | | | | | | | |

Supplementary Table 2. Results of BLAST searches of NCBI databases with DNA sequences of the of COI gene from isolated *Angiostrongylus* spp. adult worms collected from rats in Laguna and Agusan del Sur.

| **Sites/Codes** | | **Length (bp)** | **Details of the top BLAST hits** | | | | |
| --- | --- | --- | --- | --- | --- | --- | --- |
|  |  |  | **Identity** | **% Similarity** | **Query Cover** | **E-value** | **Accession Number** |
| **Laguna** | | | | | | | |
| 1 | PQ415790 | 424 | *A. cantonensis* | 100% | 95% | 0.0 | KU532144 |
| 2 | PQ415791 | 419 | *A. cantonensis* | 100% | 100% | 0.0 | MK570632 |
| 3 | PQ415792 | 434 | *A. cantonensis* | 100% | 94% | 0.0 | KU532144 |
| 4 | PQ415793 | 436 | *A. cantonensis* | 100% | 93% | 0.0 | KU532144 |
| 5 | PQ415794 | 423 | *A. cantonensis* | 100% | 95% | 0.0 | KU532144 |
| 6 | PQ415795 | 423 | *A. cantonensis* | 100% | 95% | 0.0 | KU532144 |
| 7 | PQ415796 | 423 | *A. cantonensis* | 100% | 95% | 0.0 | KU532144 |
| 8 | PQ415797 | 434 | *A. cantonensis* | 100% | 94% | 0.0 | KU532144 |
| 9 | PQ415801 | 423 | *A. cantonensis* | 100% | 94% | 0.0 | KU532144 |
| 10 | PQ415798 | 427 | *A. cantonensis* | 100% | 95% | 0.0 | KU532144 |
| 11 | PQ415799 | 418 | *A. cantonensis* | 100% | 95% | 0.0 | KU532144 |
| **Agusan del Sur** | | | | | | | |
| 12 | PQ415805 | 387 | *A. malaysiensis* | 100% | 96% | 0.0 | KU532152 |
| 13 | PQ415800 | 423 | *A. cantonensis* | 100% | 95% | 0.0 | KU532144 |
| 14 | PQ415804 | 435 | *A. malaysiensis* | 100% | 94% | 0.0 | KU532152 |
| 15 | PQ415803 | 402 | *A. malaysiensis* | 100% | 98% | 0.0 | KU532152 |
| 16 | PQ415802 | 430 | *A. malaysiensis* | 100% | 94% | 0.0 | KU532152 |
|  | | | | | | | |
